# Emergence and clonal transmission of multi-drug-resistant tuberculosis among patients in Chad

**DOI:** 10.1186/s12879-017-2671-7

**Published:** 2017-08-22

**Authors:** Awa Ba Diallo, Gedeon W. Ossoga, Geraldine Daneau, Seynabou Lo, Richard Ngandolo, Colette Diguimbaye Djaibé, Barou Djouater, Souleymane Mboup, Bouke C. de Jong, Aissatou G. Diallo, Florian Gehre

**Affiliations:** 1 Mycobacteria Unit, Bacteriology- Virology Laboratory, CHU Aristide Le Dantec, 30 Avenue Pasteur, BP 7325 Dakar, Senegal; 2 Institut de Recherche en Elevage pour le Developpement, N’Djamena, Chad; 3 Mycobacteriology Unit, Department of Biomedical Sciences, Institute of Tropical Medicine, Antwerp, Belgium; 4 Faculty of Health Sciences, Gaston Berger University, Saint Louis, Senegal; 5 Clinique Médico-Chirurgicale PROVIDENCE, N’Djamena, Chad; 6 Institut de Recherche en Santé, de Surveillance Epidemiologique et de Formation, Diamniadio, Senegal; 7 Medical Reserach Council (MRC) Unit, Fajara, Gambia

**Keywords:** *Mycobacterium tuberculosis*, MDR, Molecular characterization, Chad

## Abstract

**Background:**

Emergence of Multidrug-resistant (MDR) strains constitutes a significant public health problem worldwide. Prevalence of MDR tuberculosis from Chad is unavailable to date.

**Methods:**

We collected samples from consecutive TB patients nationwide in the seven major cities of Chad between 2007 and 2012 to characterize drug resistance and the population structure of circulating *Mycobacterium tuberculosis* complex (MTBC) strains. We tested drug sensitivity using Line Probe Assays and phenotypic drug susceptibility testing (DST) were used for second line drugs. We genotyped the isolates using spoligotype analysis and MIRU-VNTR.

**Results:**

A total of 311 cultures were isolated from 593 patients. The MDR prevalence was 0.9% among new patients and 3.5% among retreatment patients, and no second line drug resistance was identified. The distribution of genotypes suggests a dissemination of MDR strains in the Southern city of Moundou, bordering Cameroon and Central African Republic.

**Conclusion:**

Emerging MDR isolates pose a public health threat to Southern Chad, with risk to neighboring countries. This study informs public health practitioners, justifying the implementation of continuous surveillance with DST for all retreatment cases as well as contacts of MDR patients, in parallel with provision of adequate 2nd line regimens in the region.

**Electronic supplementary material:**

The online version of this article (doi:10.1186/s12879-017-2671-7) contains supplementary material, which is available to authorized users.

## Background

Tuberculosis (TB), caused by species of the *Mycobacterium tuberculosis* complex (MTBc), remains a major public health problem worldwide. Untreated, TB kills about half of the patients [1, 2]. According to the World Health Organization (WHO) 9.6 million people developed TB in 2014, while 6 million new TB patients were reported to WHO, suggesting that worldwide 37% of new patients went undiagnosed or were not reported, likely lacking appropriate treatment [3]. In 2014, 12,305 TB cases were reported in Chad, of whom 22% died, accounting for a major proportion of morbidity and death in the country [4]. Laboratories capable of performing culture or molecular DST for TB patients are still lacking in the country, and this contributes to the spreading of TB in Chad [5, 6]. The report of the National Tuberculosis Program (NTP) in 2009 stipulates a high prevalence of TB of 480/100000, although no surveys have been conducted to date [7]. The estimated prevalence of co-infection with HIV is 12%, which has increased mortality due to tuberculosis [4]. The DOTS strategy has been implemented by the NTP, and the current TB therapeutic regimen used in Chad includes 2 months of quadri-therapy with rifampicin (R), isoniazid (H), pyrazinamide (Z) and ethambutol (E), followed by 6 months with isoniazid and ethambutol (2RHZE/6HE). For retreatment patients, treatment includes 2 months of rifampicin, isoniazid, ethambutol, pyrazinamide and streptomycin followed by 1 month of rifampicin, isoniazid, pyrazinamide and ethambutol and finally 5 months of rifampicin, isoniazid and ethambutol (2RHZES/1RHZE/5RHE) [8].

Globally, the emergence of strains resistant to multiple antibiotics has compromised global TB management. According to WHO, about 480,000 cases of multi-drug resistant (MDR) TB have been reported worldwide in 2014, and nearly 9% of MDR-TB cases were extensively drug-resistant cases (XDR) [3, 9]. In Chad, as no such data on drug-resistant TB was available, we collected samples from seven major cities between 2007 and 2012 to measure drug-susceptibility to first and second line drugs, and to study the population structure of circulating strains. This study demonstrates the emergence and clonal transmission of MDR-TB strains, originating from one of two major transmission clusters of TB strains in the Southern city of Moundou, close to the Cameroonian and Central African Republic’s border.

## Methods

### Study setting

Consecutive smear positive sputum was collected across the country and culture was performed at the Mycobacteriology Unit of the Veterinary and Zootechnical Research Laboratory of Farcha in N’djamena (Chad). After shipment of isolates, spoligotyping of strains and drug-susceptibility (DST) testing for first and second line drugs was performed in the Mycobacteriology Unit of the Bacteriology-Virology Laboratory at “Hôpital Aristide Le Dantec” in Dakar, Senegal. Chad is more densely populated in the south. To arrive at estimates representative of the country, seven regions were selected: Moundou, Doba and Sarh in the south; N’djamena in the west; Bongor in the center west, Abeche in the northeast and Mongo in central Chad. This study was conducted in three phases between 2007 and 2012 (June to October 2007, July to December 2008 and April to May 2012).

### Sample collection and bacterial culture

Over 5 years, we collected twice in Ndjamena and Bongor, in 2007 and in 2008, and in Abeche in 2007 and in 2012. In 2008, we collected samples in Doba, Sarh and Moundou. The collection in Mongo was done in 2012.

A total of 593 patients suspected of having TB were included based on clinical presentation, and two sputa from each patient were collected. Sputum was preserved in Cetyl Pyridinium Chloride (CPC) (Sigma-Aldrich) and sodium chloride (Sigma-Aldrich), and transported to the laboratory in N’djamena [10] where smear microscopy was performed using Ziehl Neelsen (ZN) method and positive sputa were cultured on Lowenstein-Jensen slopes (LJ) with glycerol. Biochemical methods such as catalase test, nitrate reduction, thiophene-2 carboxylic acid hydrazide (TCH), and smooth appearance of colonies were used to differentiate MTBC and mycobacteria other than tuberculosis (MOTT).

### DNA extraction and genotyping of Mycobacterial isolates

DNA was extracted using the CTAB method as previously described by Van Embden et al. [11] and adjusted to a final concentration of 10 ng/μl in Tris-EDTA (Sigma-Aldrich) [11].

To assign lineages and families to mycobacterial isolates, spoligotyping [12] was performed, and binary codes were analyzed using the TB Insight online software (http://tbinsight.cs.rpi.edu/run_tb_lineage.html). For MDR isolates, 24 locus MIRU-VNTR was performed at Genoscreen (Lille, France) to confirm potential chains of transmission. A Neighbor-Joining tree was constructed using the MIRU-VNTRplus homepage (www.miruvntr-plus.org), incorporating genotypic data, as well as individual resistance patterns and mutations.

### Drug-susceptibility testing (DST)

For resistance testing to rifampicin and isoniazid, we used the MTBDRplus version 2 Line Probe Assay (LPA). All identified MDR isolates underwent further phenotypic and genotypic DST for second line drugs (SLD), using the Bactec MGIT 960 (Becton Dickinson) for two Fluoroquinolones (Ofloxacin 2.0 μg/ml, Moxifloxacin 2.0 μg/ml) and two injectable agents (Amikacin1.0 μg/ml, Capreomycin 2.5 μg/ml) (BD Bioscience, Becton Dickinson) [13, 14], as well as the MTBDRsl v.2 LPA (Hain Lifescience) according to the manufacturer’s instructions [15]. Strains that were suspected to be resistant to fluoroquinolones were sequenced for gyrAB (primers TAAGAGCGCCACCGACATCGGTGGATTG and GATGAAATCGACTGTCTCCTCGTCGATTTCCC for PCR, and TAAGAGCGCCACCGACATCGGTGGATTG and GTCGATTTCCCTCAGCATCTCCATC for sequencing). Resistance to pyrazinamide was analyzed by sequencing *pncA* gene (primers GGCCCGATGAAGGTGTCGTAGAAGC and CGACCTGGAAAGGCAACCCGAGAG for PCR, GGCCCGATGAAGGTGTCGTAGAAGC for sequencing). Sequencing was performed at Macrogen (The Netherlands), and sequences compared to reference DNA sequence from *Mycobacterium tuberculosis* H37Rv (PubMed accession number NC_000962, accessed May 2013) using MEGA5 software [16].

## Results

### Study population and *M. tuberculosis* strains isolated

**Fig. 1:**
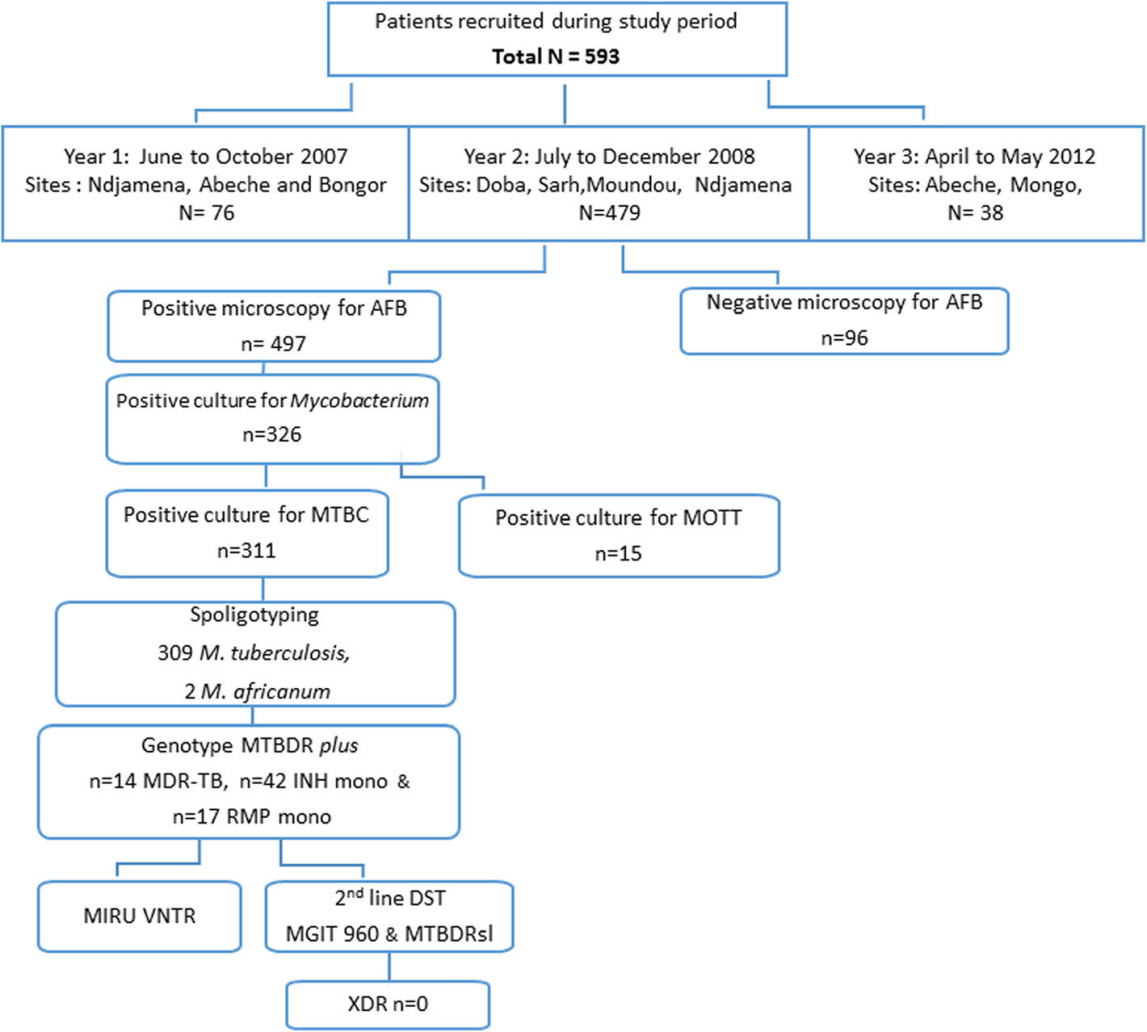
Flowchart of processed samples collected from seven major cities in Chad between 2007 and 2012. AFB (Acid Fast Bacilli), MOTT (Mycobacteria Other Than Tuberculosis), MTBc (Mycobacterium tuberculosis complex), MDR (multidrug resistant), INH mono (INH monoresistant), RMP mono (Rifampicine monoresistant)

Of the 593 samples collected, a total of 326 samples were positive after culture, and 311 were available for analysis. The 311 patients included 224 (72.0%) men and 87 (27.9%) women, between 12 and 70 years of age. The majority of the samples, 236 (75.9%), were isolated from new TB patients, and the remaining 75 (24.1%) from retreatment patients (Fig. 1).

### Genetic diversity of *M. tuberculosis* complex

**Fig. 2:**
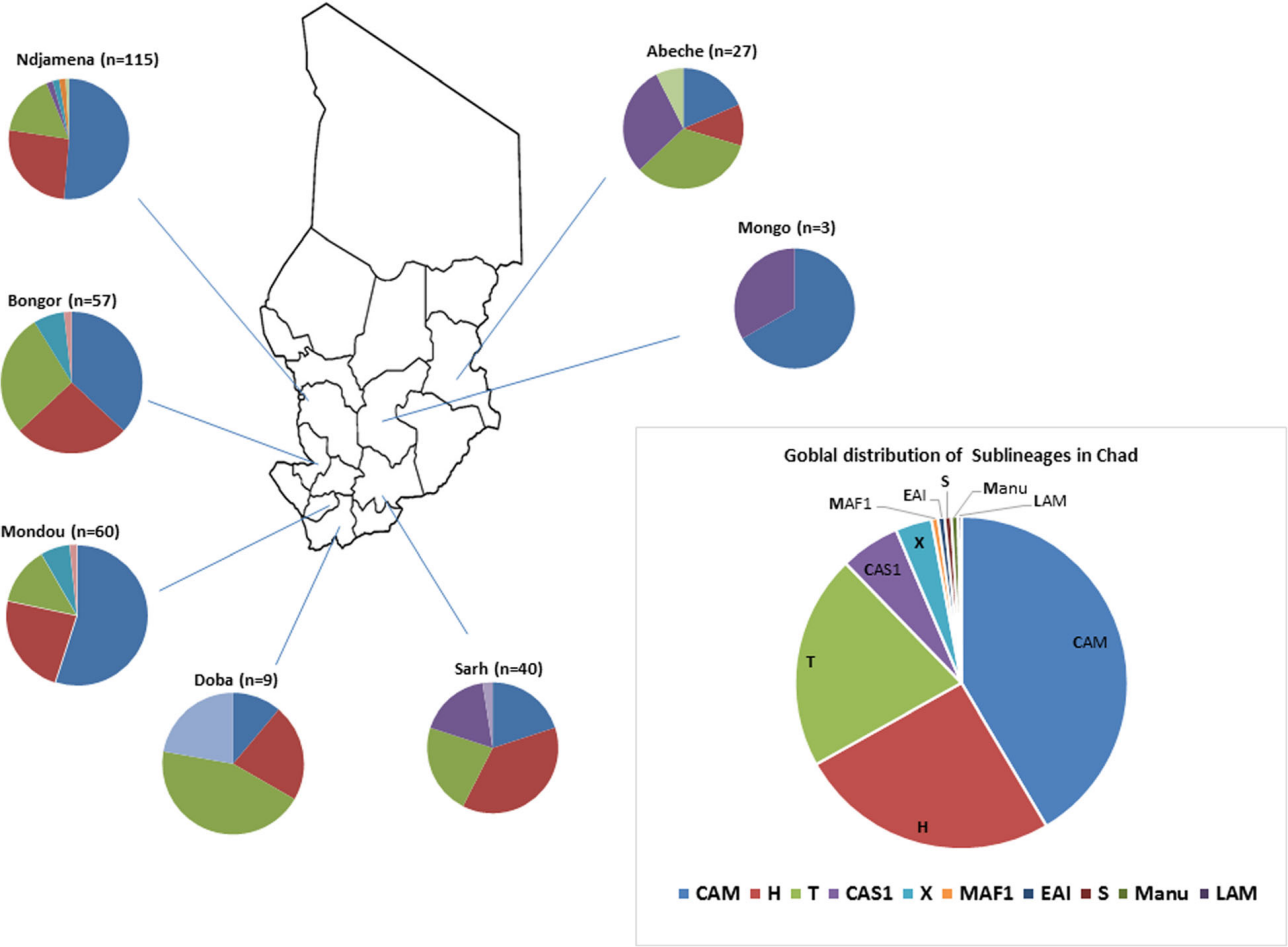
The geospatial proportional distribution of spoligotype-based mycobacterial families amongst the studied cities in Chad. Main pie charts indicate the lineage distribution. Numbers into brackets represent the number of strains

We identified 309 (99.4%) *Mycobacterium tuberculosis* sensu stricto and 2 (0.6%) *Mycobacterium africanum* West Africa 1 (MAF1, Lineage V) isolates (Additional file 1: Table S1). The majority of strains belonged to the modern Euro-American lineage IV, besides small numbers of isolates from Lineage I (2, 0.6%), Lineage III (18, 5.7%), and Lineage V (2, 0.6%). The major families within Lineage IV included LAM10_Cameroon (129, 41.8%), H1 (60, 19.2%), T1 (24, 7.7%), T2 (22, 6.7%), H3 (17, 5.4%), T1-RUS2 (9, 2.8%), X2 (7, 2.2%), T5-RUS1 (5, 1.6%). Minor families were X1, S, LAM11-ZWE, H, T, T3 and T5. The spatial distribution of the various families by city is shown in Fig. 2. We isolated *n* = 15, MOTT which were excluded from the study.

### Resistance to antituberculosis drugs

Overall, resistance to any of the two major first line drugs (rifampicin or isoniazid) was identified in 73 patients (23.4%). Rifampicin mono-resistance was identified in 5 (1.61%) new and 12 (3.8%) retreatment patients. Isoniazid mono-resistance was observed in 14 (4.5%) new and 28 (9%) retreatment patients. MDR was identified in 3 (0.9%) new and 11 (3.5%) retreatment patients. Rifampicin resistance was caused by *rpoB* gene mutations H526Y, H526D or S531 L. Regarding isoniazid resistance, 7.4% was based on the S315 T1 mutation observed in the katG only, 6.1% carried the C − 15 T mutation in *inhA* promotor only. We did not find any association for the two type of mutations observed. (Table 1). All MDR strains were tested for second line drug resistance and we found no XDR strains.

**Table 1:** Main gene mutations found among isolates conferring resistance

Gene	Probes	Codon analysed for rpoB and katG. nucleic acid position for inhA	Developing mutation band	Mutation	Resistance INH N (%) 42 (13. 50)	Resistance RIF N (%) 17 (5.47)	MDR N (%) 14 (4.50)
rpoB	WT7	526–529	rpoB MUT2A	H526Y	-	5 (1.61)	0
			rpoB MUT2B	H526D		4 (1.29)	0
	WT8	530–533	rpoB MUT3	S531 L	-	8 (2.57)	7 (2.25)
katG	WT	315	MUT1	S315 T1	23 (7.40)	-	7 (2.25)
			MUT2	S315 T2	-		
inhA	WT1	-15	MUT1	C15T	19 (6.11)	-	-

### Population structure of resistant isolates

**Fig. 3:**
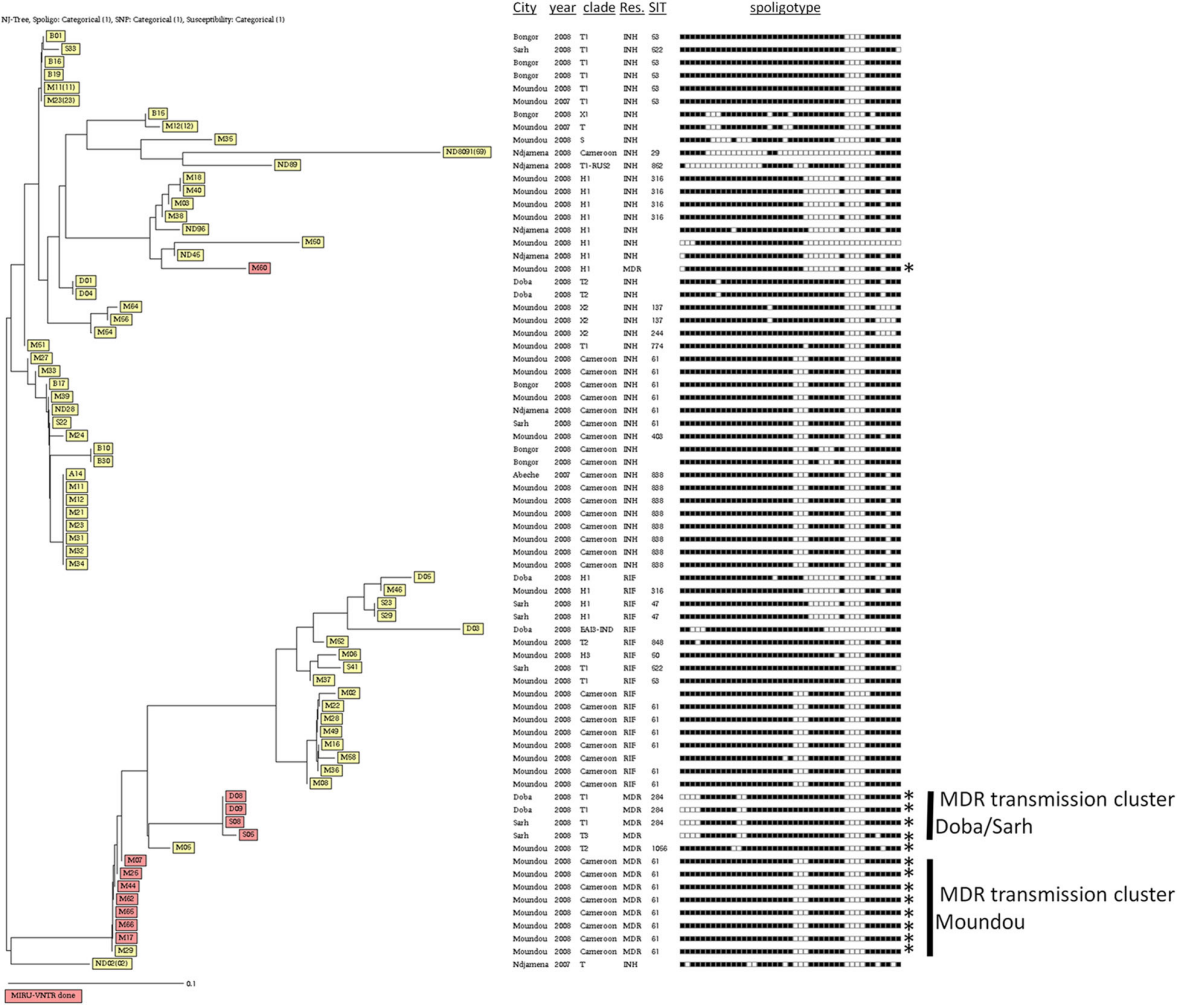
Neighbour-Joining Tree based on all isolates with any resistance to first-line drug in Chad. Individual spoligotypes, DST data and resistance-conferring mutations were taken into consideration. The asterisks (*) indicate the strains that were selected for pncA sequencing and the colour indicates all MDR for MIRU

We built a phylogenetic tree including all isolates with any resistance, based on spoligotyping, drug-susceptibility and resistance-conferring mutations (Fig. 3). When further stratifying the genotypic data by geographical origin, i.e. by city of isolation, we identified two major MDR transmission clusters, in Sarh/Doba and in Moundou respectively.

**Fig. 4:**
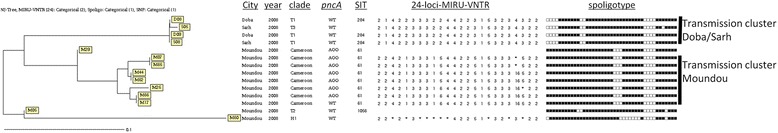
Neighbour-Joining Tree of all MDR isolates based on individual spoligo- and 24-loci MIRU-VNTR-types, Clade, Year, SIT and *pncA* results. AGG (A = Adenine, G = Guanine)

To confirm these clusters, we conducted 24-loci-MIRU-VNTR typing for these MDR isolates (Fig. 4) and found one chain of transmission in Sarh/Doba, and another chain within the Moundou cluster. Interestingly, all MDR strains carried the same resistance-conferring mutations (*rpoB* S531 L*, katG* S315 T1), as detected by MTBDR*plus*. Seven isolates belonged to SIT61 from Moundou, including three new cases, presented a same mutation in *pncA* (Del 143 AGG). Higher proportion of MDR strains was found respectively in T3 100% (1/1), T1 13% (3/24), CAM family 6% (8/130), T2 5% (1/21) and H1 1% (1/61) while no MDR was found in other lineages. The distribution of resistance patterns amongst the various lineages in Chad is displayed in Table 2.

**Table 2:** Drug resistance profile according to lineages found in Chad

Clade/Lineage	MDR		RMP mono		INH mono		Pan susceptible		Total
	N	(%)	N	%	N	%	N	%	N
Afri-2	0	(0)	0	(0)	0	(0)	2	(100)	2
Cameroon	8	(6)	8	(6)	17	(13)	97	(75)	130
CAS1	0	(0)	0	(0)	0	(0)	18	(100)	18
EAI3-IND	0	(0)	1	50	0	(0)	1	(50)	2
H1	1	(1)	4	(7)	8	(13)	48	(79)	61
H3	0	(0)	1	(6)	0	(0)	16	(94)	17
LAM11	0	(0)	0	(0)	0	(0)	1	(100)	1
Manu1	0	(0)	0	(0)	0	(0)	1	(100)	1
Manu2	0	(0)	0	(0)	0	(0)	2	(100)	2
S	0	(0)	0	(0)	1	(50)	1	(50)	2
T	0	(0)	0	(0)	2	(67)	1	(33)	3
T1	3	(13)	2	(8)	7	(29)	12	(50)	24
T1-RUS2	0	(0)	0	(0)	1	(11)	8	(19)	9
T2	1	(5)	1	(5)	2	(10)	17	(80)	21
T3	1	(100)	0	(0)	0	(0)	0	(0)	1
T5	0	(0)	0	(0)	0	(0)	2	(100)	2
T5-RUS2	0	(0)	0	(0)	0	(0)	5	(100)	5
X1	0	(0)	0	(0)	1	(33)	2	(67)	3
X2	0	(0)	0	(0)	3	(43)	4	(57)	7
Total	14	(4.5)	17	(5.4)	42	(13.5)	238	(76.6)	311

## Discussion

In this first survey of drug resistance in Chad, we identified 23.4% resistance to first line drugs for all patients, and we found respectively 0.9% and 3.5% of MDR-TB strains in new and retreatment patients. Up to now, no data was available in Chad regarding MDR and extensively drug-resistant (XDR) TB, although those strains pose real public health problems. We report here such results for the first time. Several strains had the same spoligotype pattern and same resistance mutations to rifampicin and isoniazid, suggestive of two chains of transmission of MDR strains (see Fig. 3). As spoligotype analysis alone does not have sufficient resolution to identify chains of transmission, we re-typed MDR strains using high-resolution 24 loci MIRU-VNTR-typing, and added the genotypic profile of the *pncA* gene. In our study population, 4.5% of patients were infected with MDR-TB strains and we found several genotypically identical MDR isolates suggestive of two ongoing transmission chains of MDR strains in the towns of Moundou and between Doba and Sarh. Some of those patients were new cases (3, 0.9%). These findings suggest that MDR strains are present in Chad, but also being transmitted. In consequence, TB control measures should include the rapid implementation of continuous surveillance of rifampicin resistance in retreatment patients nationwide, as recommended by WHO, with second line resistance testing when rifampicin resistance is identified, and the availability of effective therapy for resistant TB.

In respect to the population structure of the MTBc, we observed that the Cameroon family SIT61 within Lineage 4, was the most frequent. These CAM genotypes were isolated for the first time in Cameroon by Niobe-Eyangoh et al. [17]. Our study is in line with findings by Diguimbaye et al. in 2006 [18] from the Chari-Baguirmi region of Chad that reported that 33% belonged to the CAM family. Our study has identified the CAM family strains in all major cities of Chad, with highest prevalence found in N’djamena (18.9%), Moundou (10.6%) and Bongor (6.7%), which border with Cameroon. As the CAM isolates were described to be highly transmissible and were associated with an increased risk of developing drug-resistance, it is advisable to monitor the longitudinal spread of these strains in Chad.

Limitations of this study include the potential selection bias, as we did not apply a formal drug resistance survey design with cluster representative sampling. Moreover, the prevalence of resistance may be underestimated due to the use of the LPA, which may have missed some rifampicin resistance (especially *rpoB* mutations at the positions 511, 533, and/or 572), and is only 90% sensitive for isoniazid resistance.

## Conclusion

In conclusion, the MDR strains isolated in patients in the towns of Moundou, Sarh and Doba occur in two genotypic clusters, suggesting that most resistant TB is due to ongoing transmission. Therefore, our findings suggest that priorities for TB control in Chad should include the early diagnosis and effective treatment of MDR-TB patients, with provision for rapid second line DST testing and availability of treatment options for potential future XDR-TB patients, especially in the south of the country.

## Additional file

Additional file 1: Table S1. Individual spoligotyping data from isolates collected in Chad. a: The black and white boxes indicate the presence and absence, respectively, of the specific spacer at positions 1–43 in the DR locus; b: Lineage designations according to SITVIT2 using revised SpolDB4 rules; c: Clustered strains correspond to a similar spoligotype pattern shared by 2 or more strains “within this study”; as opposed to unique strains harboring a spoligotype pattern that does not match with another strain from this study. (DOCX 19 kb)

## Abbreviations

AFB: Acid fast bacilli
CPC: Cetyl pyridinium chloride
CTAB: Cetyl tri-methyl ammonium bromide
DST: Drug sensitivity testing
E: Ethambutol
H: Isoniazid
HIV: Human Immunodeficiency virus
LJ: Lowenstein Jensen
MDR: Multiple drug resistance
MOTT: Mycobacteria other than tuberculosis
MTBc: *Mycobacterium tuberculosis complex*
NTP: National Tuberculosis Program
R: Rifampicin
SIT: Shared International type
SLD: Second line drug
TB: Tuberculosis
XDR: Extensively drug resistant
Z: Pyrazinamide
ZN: Ziehl Nielsen
