# Supplementary material for: Emergence and clonal transmission of multi-drug-resistant tuberculosis among patients in Chad
Source: BMC Infect Dis. 2017 Aug 22;17:579. doi: 10.1186/s12879-017-2671-7 (PMC5567628; doi:10.1186/s12879-017-2671-7)
Supplement: Additional file 1: Table S1. — Individual spoligotyping data from isolates collected in Chad. a: The black and white boxes indicate the presence and absence, respectively, of the specific spacer at positions 1–43 in the DR locus; b: Lineage designations according to SITVIT2 using revised SpolDB4 rules; c: Clustered strains correspond to a similar spoligotype pattern shared by 2 or more strains “within this study”; as opposed to unique strains harboring a spoligotype pattern that does not match with another strain from this study. (DOCX 19 kb) [file 12879_2017_2671_MOESM1_ESM.docx]

**Table S1.** Identification of sub-types of *M. tuberculosis* complex in SITVIT database.

| Spoligotype description^a^ | octal Number | Nb. in  study (%) | Lineage^b^ | Clustered vs. Unique^c^ |
| --- | --- | --- | --- | --- |
| ■■■■■■■■■■■■■■■■■■■■■■□□□■■■■■■■□□□□■■■■■■■ | 777777743760771 | 82 (26.3) | Cameroon | Clustered |
| ■■■■■■■■■■■■■■■■■■■■■■□□□■■■■■■■□□□□■■■■□■■ | 777777743760751 | 15 (4.8) | Cameroon | Clustered |
| □□■■■■■■■■■■■■■■■■■■■■□□□■■■■■■■□□□□■■■■■■■ | 177777743760771 | 13 (3.8) | Cameroon | Clustered |
| ■■■■■■■■■■■■■■■■■■□□■■□□□■■■■■■■□□□□■■■■■■■ | 777777143760771 | 5 (1.6) | Cameroon | Clustered |
| ■■■■■■■■■■■■■■■■■■■■■■□□□■■■■■□□□□□□□□■■■■■ | 777777743700171 | 4 (1.2) | Cameroon | Clustered |
| ■■■■■■■■■■■■■■■■■■■■■■□□□■■□□□■■□□□□■■■■■■■ | 777777743060771 | 2 (0.6) | Cameroon | Clustered |
| ■■■■■■■■■■■■■■■■■■□■■■□□□■■■■■■■□□□□■■■■■■■ | 777777343760771 | 2 (0.6) | Cameroon | Clustered |
| ■■■■■■■■■■■■■■■■■■■■■■□□□■■■■■■■□□□□□■■■■■■ | 777777743760371 | 2 (0.6) | Cameroon | Clustered |
| ■■■■■■■■■■■■■■■■■■■■■■□□□■■■■■■■□□□□■■■□■■■ | 777777743760731 | 1 (0.3) | Cameroon | Unique |
| ■■■■■■□□■□□■■□□■■■■■□■□□□■■■■■■■□□□□■■□■■□■ | 771147643760661 | 1 (0.3) | Cameroon | Unique |
| ■■■■■■■■■□■■■■■■■■■■■■□□□□□■■■■■□□□□■■■■■■■ | 777377740760771 | 1 (0.3) | Cameroon | Unique |
| ■■■■■■■■■■■■■■■■■■■■□■□□□■■■■■■■□□□□■■■■■■■ | 777777643760771 | 1 (0.3) | Cameroon | Unique |
| ■■□□□□□■■■■■■■■■■■■■■■□□□■■■■■■■□□□□■■■■■■■ | 603777743760771 | 1 (0.3) | Cameroon | Unique |
| ■■■■■■■■■■■■■■■■■■■■■■■■□□□□□□□■□□□□■■■□■■■ | 777777770020731 | 20 (6.4) | H1 | Clustered |
| ■■■■■■■■■■■■■■■■■■■■■■■■■□□□□□□■□□□□■■■■■■■ | 777777774020771 | 11 (3.5) | H1 | Clustered |
| ■■■■■■■■■■■■■■■■■■■■■■■■□□□□□□□□□□□□□□□□□□□ | 777777770000000 | 6 (1.9) | H1 | Clustered |
| ■■■■■■■■■■■■□□□■■■■■■■■■□□□□□□□■□□□□■■■□■■■ | 777707770020731 | 4 (1.2) | H1 | Clustered |
| □■■■■■■■■■■■■■■■■■■■■■■■□□□□□□□■□□□□■■■□■■■ | 377777770020731 | 4 (1.2) | H1 | Clustered |
| □□□■■■■■■■■■■■■■■■■■■■■■□□□□□□□□□□□□□□□□□□□ | 077777770000000 | 4 (1.2) | H1 | Clustered |
| ■■■■■■■■□□□□■■■■■■■■■■■■■□□□□□□■□□□□■■■■■■■ | 776077774020771 | 2 (0.6) | H1 | Clustered |
| ■■■■■■■■■■■■□■■■■■■■■■■■□□□□□□□■□□□□■■■□■■■ | 777737770020731 | 2 (0.6) | H1 | Clustered |
| ■■■■■■■■■■■■■■■■■□■■■■■■□□□□□□□□□□□□□□□□□□□ | 777776770000000 | 1 (0.3) | H1 | Unique |
| ■■■■■■■■■■■■■■■■■■■■■■■■□□□□□□□■□□□□■■□□■■■ | 777777770020631 | 1 (0.3) | H1 | Unique |
| ■■■■■■■■■■■■■□■■■■■■■■■■□□□□□□□■□□□□■■■□■■■ | 777757770020731 | 1 (0.3) | H1 | Unique |
| ■■■■■■■■■■■■■■■■■■■□■■■■□□□□□□□■□□□□■■□□■■■ | 777777570020631 | 1 (0.3) | H1 | Unique |
| ■■■■■■■■■■■■■■■■■■□■■■■■□□□□□□□■□□□□■■□□■■■ | 777777370020631 | 1 (0.3) | H1 | Unique |
| ■■■■■■■■■■■■■■■■■■■■□■■■□□□□□□□□□□□□□□□□□□□ | 777777670000000 | 1 (0.3) | H1 | Unique |
| ■■■■■■■■■■□■■■■■■■■■■■■■■□□□□□□■□□□□■■■□■■■ | 777577774020731 | 1 (0.3) | H1 | Unique |
| ■■■■■■■■■■■■■■■■■■■■■■■■■■■■■■■■□□□□■■■■■■■ | 777777777760771 | 6 (1.9) | T1 | Clustered |
| ■■■□■■■■■■■■■■■■■■■■■■■■■■■■■■■■□□□□■■■■■■■ | 737777777760771 | 6 (1.9) | T1 | Clustered |
| ■■■■■■■■■■■■■■■■■■■■■■■■□■■■■■■■□□□□■■■■■■■ | 777777773760771 | 5 (1.6) | T1 | Clustered |
| □□□□■■■■■■■□□■■■■■■■■■■■■■■■■■■■□□□□■■■■■■■ | 037637777760771 | 3 (0.9) | T1 | Clustered |
| ■■■■■■■■■■■■■■■■■■■■■■■■■■■■■■■■□□□□■■■■■■□ | 777777777760770 | 3 (0.9) | T1 | Clustered |
| ■■■■■■■■■■■■■■■■■■■■■■□□□□■■■■■■□□□□■■■■■■■ | 777777741760771 | 1 (0.3) | T1 | Unique |
| ■■■■■■■■■■□□■■■■■■■■■■■■■■■■■■■■□□□□■■■□■■■ | 777477777760731 | 5 (1.6) | T2 | Clustered |
| ■■■■■■■■■■■■■■■■■■■■■■■■■■■■■■■■□□□□■■■□■■■ | 777777777760731 | 4 (1.2) | T2 | Clustered |
| ■■■□■■■■■■■■■■■■■■■■■■■■■■■■■■■■□□□□■■■□■■■ | 737777777760731 | 4 (1.2) | T2 | Clustered |
| ■■■■■■■■■■■■■■■■■■■■■■■■■■■■■■■■□□□□■■■□■■□ | 777777777760730 | 1 (0.3) | T2 | Unique |
| ■■■■■■■■■■■■■■■■□■■■■■■■■■■■■■■■□□□□■■■□■■■ | 777775777760731 | 1 (0.3) | T2 | Unique |
| ■■■■■□□□□■■■■■□□■■■■■■■■■■■■■■■■□□□□■■■□■■■ | 760763777760731 | 1 (0.3) | T2 | Unique |
| ■■■■■□■■□■■■■■■■■■■■■■■■■■■■■■■■□□□□■■■□■■■ | 766777777760731 | 1 (0.3) | T2 | Unique |
| ■■■■■■■■■■□□■□■■■■■■■■■■■■■■■■■■□□□□■■■□■■■ | 777457777760731 | 1 (0.3) | T2 | Unique |
| ■■■■□■■□■■■■■■■■■■■■■■■■■■■■■■■■□□□□■■■□■■■ | 755777777760731 | 1 (0.3) | T2 | Unique |
| ■■■■■■■■■■□□□■■■■■■■■■■■■■■■■■■■□□□□■■■□■■■ | 777437777760731 | 1 (0.3) | T2 | Unique |
| ■■■■■■■□■■■■■■■■■■■■■■■■■■■■■■■■□□□□■■■□■■■ | 775777777760731 | 2 (0.6) | T2 | Clustered |
| ■■■■■■■■■■■■■■■■■■■■■■■■■■■■■■□■□□□□■■■■■■■ | 777777777720771 | 5 (1.6) | H3 | Clustered |
| ■■■■■■■■■■■■■■■■■■■■■■■■■■■■■■□■□□□□■■■□□■■ | 777777777720711 | 4 (1.2) | H3 | Clustered |
| ■■■■■■■■■■■■■■■■■■■■■■■■■■■■□■□■□□□□■■■□■■■ | 777777777520731 | 3 (0.9) | H3 | Clustered |
| ■■■■■■■■■■■■■■■■■■■□■■■■□□□□□□□■□□□□■■■□■■■ | 777777570020731 | 2 (0.6) | H3 | Clustered |
| ■■■■■■■■■■■■■■■□■■■■■■■■■■■■■■□■□□□□■■■□■■■ | 777773777720731 | 1 (0.6) | H3 | Unique |
| ■■■■■■■■■■■■■■■■■■■■■■■■■■■■■■□■□□□□■■□□□■■ | 777777777720611 | 1 (0.3) | H3 | Unique |
| ■■■■■■■■■■■■■■■■■■■■■■■■■■■■□■□■□□□□■■■□■■■ | 777777777520731 | 1 (0.3) | H3 | Unique |
| ■■■□□□□■■■■■■■■■■■■■■■□□□□□□□□□□□□■■□□■■■■■ | 703777740003171 | 12 (3.8) | CAS1-Delhi | Clustered |
| ■■■□□□□■■■■■■■■■■■■■■■□□□□□□□□□□□□■■■■■■■■■ | 703777740003771 | 2 (0.6) | CAS1-Delhi | Clustered |
| ■■■□□□□■■■■■■■■■■■■■■■□□□□□□□□□□□□□□□□■■■■■ | 703777740000171 | 2 (0.6) | CAS1-Delhi | Clustered |
| ■■□□□□□■■■■■■■■■■■■■■■□□□□□□□□□□□□■■□□■■■■■ | 603777740003171 | 2 (0.6) | CAS1-Delhi | Clustered |
| ■□□□□□□□□□□□□□□□■■■■■■□□□■■■■■■■□□□□■■■■■■■ | 400003743760771 | 8 (2.5) | T1-RUS2 | Clustered |
| ■□□□□□□□□□□□□□□□□□□□□□□□■■■■■■■■□□□□■■■□■■■ | 400000007760731 | 1 (0.3) | T1-RUS2 | Unique |
| ■■■■■■■■■■■■■■■■■□■■■■■■■■■■■■■■□□□□■■□□□□■ | 777776777760601 | 4 (1.2) | X2 | Clustered |
| ■■■■■■■■■■■■■■■■■■■■■■■■■■■■■■■■□□□□■■□□□□■ | 777777777760601 | 3 (0.9) | X2 | Clustered |
| ■■■■■■■■■■■■■□□□□■□□□□□■■■■■■■■■□□□□■■■■■■■ | 777741017760771 | 2 (0.6) | T5-RUS1 | Clustered |
| ■■■■■■■■■■■■■□□□□□□□□□□■■■■■■■■■□□□□■■■■■■■ | 777740017760771 | 1 (0.3) | T5-RUS1 | Unique |
| ■■■■■■■■■■■□■□□□□□□□□□□■■■■■■■■■□□□□■■■■■■■ | 777640017760771 | 1 (0.3) | T5-RUS1 | Unique |
| ■■■■■■■■■■■■■□■□□□□□□□□■■■■■■■■■□□□□■■■■■■■ | 777750017760771 | 1 (0.3) | T5-RUS1 | Unique |
| ■■■■■□□□□■■■■□□□■■■■■■■■■■■■■■■■□□□□■■■□■■■ | 760743777760731 | 1 (0.3) | T | Unique |
| ■□■■■■■■■■■■■□□■■■■■□■□□□■■■■■■■□□□□■■□■■□■ | 577747643760661 | 1 (0.3) | T | Unique |
| ■■■■■□□□■■■■■■■■■□■■□□■■■■■■■■■■□□□□■□■■■■■ | 761776637760571 | 1 (0.3) | T | Unique |
| ■■■■■□□□■■■■■■■■■□■■■□■■■■■■■■■■□□□□■□■■■■■ | 761776737760571 | 3 (0.9) | X1 | Clustered |
| ■■■■■■■□□□□□□■■■■■■■□□□□■■■■■■■■■■■■□□□■■■■ | 774037607777071 | 2 (0.6) | AFRI_1 | Clustered |
| ■■□□□■■■■■■■■■■■■■■■■■■■■■■■□□□□□□□□□□□□■■■ | 617777777400031 | 2 (0.6) | EAI3-IND | Clustered |
| ■■■■■■■■■■■■■■■■■■■■■■■■■■■■■■■■□□■■■■■■■■■ | 777777777763771 | 2 (0.6) | Manu2 | Clustered |
| ■■■■■■■■■■■■■■■■■■■■■■□■■■■■■■■■□□□□■■■■■■■ | 777777757760771 | 2 (0.6) | T5 | Clustered |
| ■■■■■□□□□□□□□□□□□■■□□□□□□□□□□□□□□□□□□□■■■■■ | 760001400000171 | 1 (0.3) | H | Unique |
| ■■□□■■■■■■■■■■■■■■■□□□□□□□□□□□□■□□□□■■■□■■■ | 637777400020731 | 1 (0.3) | LAM11-ZWE | Unique |
| ■■■■■□□□□□■■■■■□□■■■■■■■■■■■■■■■□□□□■■■□■■■ | 760371777760731 | 1 (0.3) | S | Unique |
| ■■■■■■□□□□■□□□■■■■■■□■■■■■■■■■□□□□□□■■■□■■■ | 770217677700731 | 1 (0.3) | S | Unique |
| □□□□■■■■■■■□□■■■■■■■■■■■■■■■■■■■□□□□■■□■■■■ | 037637777760671 | 1 (0.3) | T3 | Unique |
|  |  |  |  |  |
| a: The black and white boxes indicate the presence and absence, respectively, of the specific spacer at positions 1-43 in the DR locus  b: Lineage designations according to SITVIT2 using revised SpolDB4 rules;  c: Clustered strains correspond to a similar spoligotype pattern shared by 2 or more strains “within this study”; as opposed to unique strains harboring a spoligotype pattern that does not match with another strain from this study | | | | |
